# Loss of *Malat1* does not modify age- or diet-induced adipose tissue accretion and insulin resistance in mice

**DOI:** 10.1371/journal.pone.0196603

**Published:** 2018-05-10

**Authors:** Sophie Carter, Stéphanie Miard, Louise Boivin, Sandrine Sallé-Lefort, Frédéric Picard

**Affiliations:** 1 Centre de recherche de l'Institut universitaire de cardiologie et de pneumologie de Québec, QC, Canada; 2 Faculty of Pharmacy, Laval University, Québec, QC, Canada; Vall d'Hebron Institut de Recerca, SPAIN

## Abstract

Several studies have suggested that signals emerging from white adipose tissue can contribute to the control of longevity. In turn, aging is associated with perturbed regulation and partitioning of fat depots and insulin resistance. However, the exact mechanisms involved in these relationships remain undetermined. Using RAP-PCR on adipose tissue of young and old male mice coupled with qPCR validation, we have uncovered the long non-coding RNA *Malat1* as a gene robustly downregulated in visceral white adipose tissue (vWAT) during normal aging in male mice and men. Reductions in *Malat1* expression in subcutaneous WAT (scWAT) were also observed in genetic (*ob* and *db*) as well as diet-induced models of obesity. Based on these findings, *Malat1*+/+ and *Malat1*-/- mouse littermates were thus probed to detect whether loss of *Malat1* would impact age or diet-induced gain in fat mass and development of glucose intolerance. Contrary to this hypothesis, male and female *Malat1*-deficient mice gained as much weight, and developed insulin resistance to a similar extent as their *Malat1*+/+ littermates when studied up to eight months old on regular chow or a high-fat, high-sucrose diet. Moreover, we observed no marked difference in oxygen consumption, food intake, or lipid profiles between *Malat1*+/+ and *Malat1*-/- mice. Therefore, we conclude that the overall metabolic impact of the absence of *Malat1* on adipose tissue accretion and glucose intolerance is either physiologically not relevant upon aging and obesity, or that it is masked by as yet unknown compensatory mechanisms.

## Introduction

A well-known consequence of aging is a redistribution of adipose tissue, favoring accumulation in the intra-abdominal visceral depot at the detriment of subcutaneous depots. This reconfiguration has been linked to the development of a central and peripheral resistance to the effects of insulin, as well as profound alterations in lipoprotein-lipid profile [1]. Moreover, age-associated infiltration of cells from the immune system and changes in adipokine production trigger a pro-inflammatory state in which differentiation of new adipocytes from mesenchymal precursor cells is impaired, further hindering insulin sensitivity [2]. With the current shift in demographics towards aging population in developed countries, these conditions likely contribute to the persistent rise in the prevalence of type 2 diabetes.

In turn, genetic interventions specifically designed to result in modifications in adipose tissue mass and biology have been associated with changes in mean and maximal longevity in many species, including mammals [3, 4]. The increase in longevity due to a reduction in total adipose tissue mass could be due to many factors, including changes in adipokine secretion, diminished infiltration of immune cells, and lowered inflammation [1, 5]. However, visceral adipose tissue could also express specific genes that ultimately shorten lifespan. Thus, aging and energy metabolism in adipose tissue have clear interconnections, which have however yet to be fully understood.

In this context, we aimed at discovering genes modulated in adipose tissue upon aging in a conserved manner, with the hypothesis that they would in turn influence age-induced alterations in energy metabolism. To unravel these genes, a non-biased, whole-genome RNA arbitrarily primed-polymerase chain reaction (RAP-PCR) assay was performed on epididymal adipose tissue (vWAT) from male C57BL/6J mice aged 4, 12 and 24 months. We notably found that the expression of *Metastasis Associated Lung Adenocarcinoma Transcript 1* (*Malat1*) was robustly reduced upon aging in mouse and human visceral WAT.

*Malat1* is a nuclear 7 kb long non-coding RNA (lncRNA) cleaved at its 3’ end, creating a 61-nt cytoplasmic by-product named *Malat1-associated small cytoplasmic RNA* (*mascRNA*) [6, 7]. Whereas the role of the *mascRNA* remains unknown, *Malat1* has been proposed to affect cellular proliferation and migration by acting as a molecular sponge on many miRNA [8–11]. Increased *Malat1* expression has been associated with cancer cell progression *in vitro* and in xenograft models [12–14]. More recently, it was shown to promote lipid accumulation in hepatocytes by altering SREBP-1c stability [15]. Yet, *Malat1*-deficient mice develop normally and show no overt phenotype, as demonstrated by four independent groups [16–20].

Based on the finding of a reduction of *Malat1* levels in adipose tissue upon aging, we hypothesized that the loss of *Malat1* would impact age or diet-induced gain in fat mass and development of glucose intolerance. Unexpectedly, the metabolic adaptations of whole-body *Malat1*-deficient mice (*Malat1*-/-) to aging or high-fat feeding were similar to those of their *Malat1*+/+ littermates. Thus, loss of *Malat1* has no apparent significant consequence on age-associated metabolic diseases.

## Materials and methods

### Animals

Male and female C57BL/6J mice of 4, 12 and 24 months old were purchased from the National Institute of Aging (NIA, USA) and the Quebec Network of Research on Aging (RQRV) colony. Male *ob/ob*, and *db/db* mice, as well as their wild-type counterparts, were from The Jackson Laboratory (Bar Harbor, ME, USA). The generation of *Malat1*+/+ and *Malat1*-/- mice on a pure C57BL/6J genetic background has been described previously [16]. In a first study, *Malat1*+/+ and *Malat1*-/- littermates were fed *ad libitum* with regular chow (Harlan NIH31) until sacrifice. In a second study, *Malat1*+/+ and *Malat1*-/- littermates were fed *ad libitum* with regular chow until reaching 11 weeks of age, and fed an obesity-inducing 45% high-fat /35% sucrose diet (Research Diets #D12451) until sacrifice. All mice had *ad libitum* access to water, and were housed individually under a 12:12 dark:light cycle and an ambient temperature of 23 ± 1°C. All mice were cared for and handled in accordance to the *Canadian Guide for the Care and Use of Laboratory Animal* and the Laval University Institutional Animal Care Committee approved the protocols. Mice were sacrificed by terminal cardiac puncture under anesthesia after an overnight fast. Harvested tissues were weighed and then snap frozen into liquid nitrogen until further processing.

### RAP-PCR

RAP-PCR was performed as described [21]. Epididymal white adipose tissue was harvested from male mice of 4, 12 and 24 months old after an overnight fast. Total RNA was extracted by the guanidinium-thiocyanate method [22]. Purity, degradation state and concentration of the RNA samples were analyzed by automated electrophoresis (Experion System, Bio-Rad). One μg of total RNA was used for first-strand synthesis at 42°C for one hour with first strand arbitrary primer (A1 to A5) in a total volume of 20 μL. One μL of this reaction was used for second-strand synthesis with radio-labeled 33^P^-ATP and equal amounts of chosen first strand arbitrary primer and a second arbitrary primer in a 50 μL total volume. Primers used for RAP-PCR are shown in S1 Table. Use of primers A1 to A5 resulted in 20 possible primer pairs. Two μL of each PCR products were separated on a sequencing gel (6% polyacrylamide/bis-acrylamide 19:1, 8M urea, 45 mM Tris-borate pH8, 1 mM EDTA in TBE buffer). Bands differentially expressed between age groups were visualized on an autoradiogram, excised from the gel, and eluted in water. Eluted DNA was then re-amplified using the same primer couple used in the second strand synthesis. Re-amplified products were isolated, cloned into TOPO vector (Invitrogen) according to manufacturer’s instructions, and sequenced on a Coulter CEQ 8000 system (Beckman).

After removal of duplicates, genes were selected on the basis of novelty and extent of modulation upon aging. Variation in expression was then confirmed by qPCR in tissues from mice of a second cohort (RQRV) as well as in adipocytes and cells from the stroma-vascular fraction (SVF) isolated from vWAT by the collagenase method as described by Rodbell [23].

Conserved modulation in the expression of selected hits was tested in omental and subcutaneous WAT from obese men obtained during ongoing bariatric surgery. These men were 18.8 to 65.8 years old (body mass index (BMI) 55.1 ± 11.4 kg/m^2^) and were divided according to age groups with averages of 23, 40, and 59 years old ± 1 year. Full patient characteristics have been described previously [24]. Briefly, none of the subjects were diabetic but ten had hypertension (and were on anti-hypertensive therapy) and four had dyslipidemia and were treated with a statin. Five subjects were smokers. Because subjects with these characteristics were evenly distributed across age groups, excluding those subjects from the analyses did not alter the present results. Approval was obtained from the medical ethics committee of the IUCPQ. All patients provided written informed consent before their inclusion in the study.

### qPCR

Quantitative PCR was carried using an ABI 7900 (Life Technologie). Chemical detection of the PCR products was achieved with SYBR Green Jumpstart Taq ReadyMix without MgCl_2_ (Sigma) as described [25]. All reactions were performed in duplicate and relative level of gene expression was determined via a standard curve composed of a mix from all cDNA. Data were corrected by the expression of a housekeeping gene whose expression remained unchanged upon aging, i.e. ß-actin (for mouse tissues) and L27 (for human WAT). Primers used are listed in S2 Table.

### Dual energy X-ray absorptiometry (DEXA) scan

Mice were anesthetized using isoflurane and placed in a calibrated Lunar PIXImus 2 (General Electric). Lean mass, fat mass, bone mineral density and % fat mass were quantified by computerized absorptiometry analysis.

### Indirect calorimetry (oxygen consumption)

Mice were placed in metabolic chambers (AccuScan Instruments, Colombus, OH) and were acclimated for 72 hours with free access to food and water. Then, oxygen consumption (VO_2_) and carbon dioxide production (VCO_2_) were measured every 15 min over 24 hours.

### Histology

Pieces of vWAT and scWAT were fixed in 4% PFA and embedded in paraffin. Sections of 4 μm were stained with hematoxylin and eosin and recorded on an Olympus BX51 microscope. Adipocyte size was analyzed with Image ProPlus 6.0 software on five images of the same dimension per depot per mouse, two mice per genotype.

### Plasma bioassays

Blood was collected from the saphenous vein into a tube containing EDTA, and plasma was stored at -80°C. Non-esterified fatty acids (NEFA), cholesterol, triglycerides and glucose were measured by colorimetric assays (RANDOX). Plasma insulin levels were quantified by ELISA (ALPCO).

### Glucose and insulin tolerance tests

Glucose tolerance was evaluated in 12 hours-fasted mice injected intraperitoneally with 2 g/kg of D-glucose. Insulin sensitivity was evaluated in 5 hours-fasted mice injected with 0.75 U/kg of insulin (Novolin, Novo Nordisk). For both tests, at different intervals before and after injections, glycemia was measured in blood from the tail vein using an Accu-Chek glucometer (Roche).

### Statistical analysis

Data are presented as mean ± S.E.M. Statistical differences were analyzed by one way, two-way, and repeated measures ANOVA, as appropriate (Statview, SAS Institute). A *p* value < 0.05 was considered significant.

## Results

**Fig 1:**
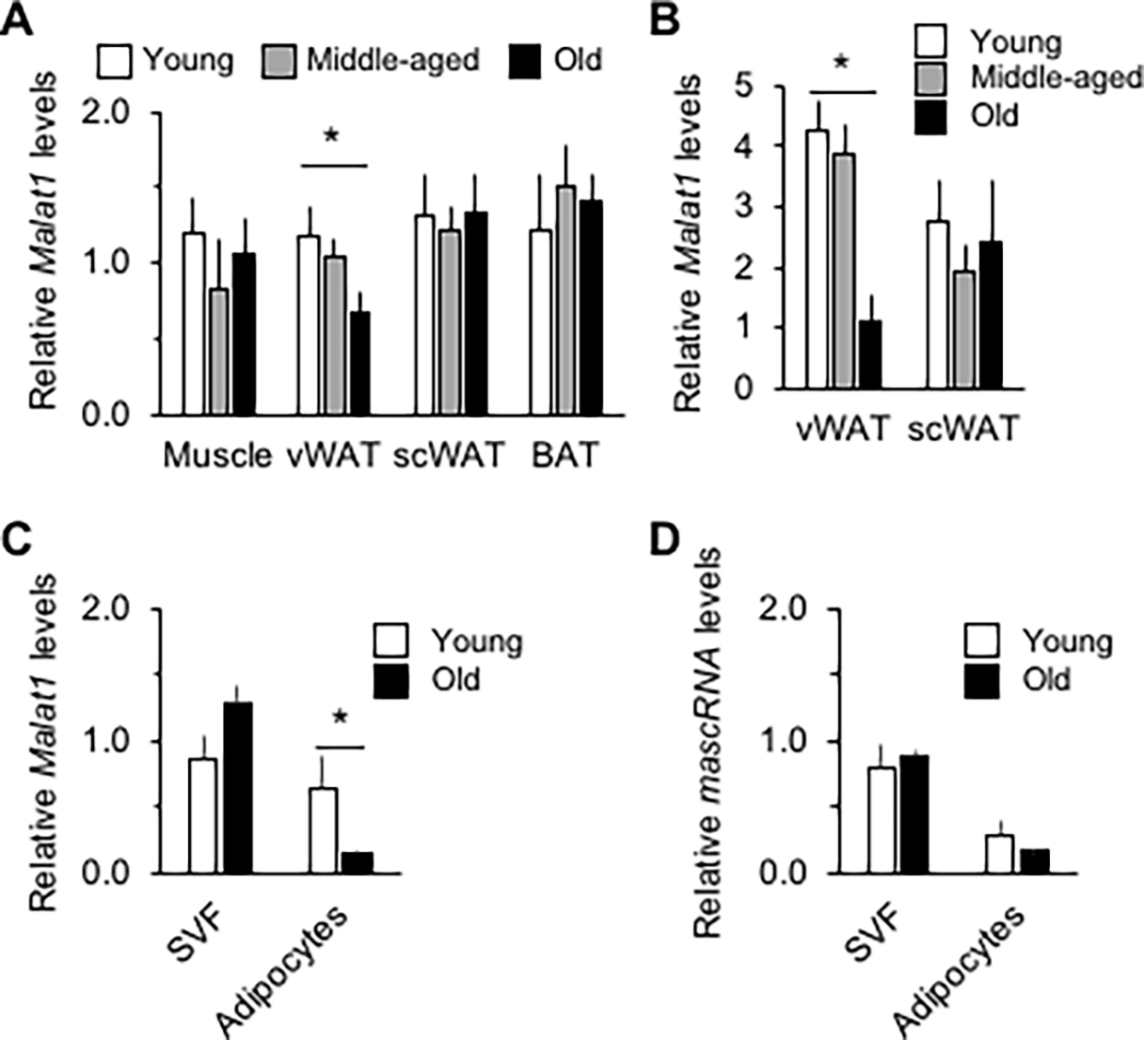
Aging is associated with a reduction in *Malat1* levels in visceral adipose tissue in mice and men. (A) Expression levels of *Malat1* in skeletal muscle, epididymal (visceral—vWAT) inguinal (subcutaneous—scWAT) white adipose tissue, and interscapular brown adipose tissue (BAT) of 4, 12 or 24 months old male C57BL/6J mice (n = 12–14). * indicates a significant difference compared with the 4 months old group (*p* < 0.05). (B) Expression levels of *Malat1* in visceral (omental) and subcutaneous white adipose tissue of men on average 23 (young), 40 (middle-aged), or 59 (old) ± 1 years old (n = 7–10). * indicates a significant difference compared with younger group (*p* < 0.05). (C-D) *Malat1* (C) and *mascRNA* (D) expression levels in cells of the stroma vascular fraction (SVF) or adipocyte freshly collagenase-isolated from vWAT of young and old male mice described in A. n = 3. * indicates a significant difference compared with the 4 months old group (*p* < 0.05).

To uncover genes that are modulated upon aging in vWAT, a RAP-PCR screening was implemented using the epididymal adipose depot of 4, 12 and 24 months old male C57BL/6J mice (strategy illustrated in S1 Fig). After cloning and sequencing of bands with the most visible changes (see S3 Table for a list of these genes), *Malat1* was revealed as a gene highly down-regulated upon aging, which was then arbitrarily chosen for further study based on novelty and lack of available annotated and/or published functions related to the regulation of energy metabolism at this time. This finding was confirmed by qPCR in a second cohort of aging mice, which quantitatively determined a significant 25% diminution in expression of *Malat1* in this depot, but no change in scWAT, gastrocnemius skeletal muscle, or interscapular brown adipose tissue (BAT) (Fig 1A). In female mice, age rather induced a diminution in *Malat1* levels in the scWAT depot (S2A Fig). In men, a robust reduction in *MALAT1* expression levels upon aging was similarly observed in omental vWAT but not scWAT (Fig 1B). Digestion of adipose depots by collagenase was then performed to identify cell populations within which *Malat1* levels were modulated. We found that, in male mice, adipocytes and SVF fractions from vWAT and scWAT expressed similar levels of *Malat1* (S2B Fig), but that, in men, its levels in adipocytes were lower than those in the SVF (S2C Fig). Interestingly, in vWAT of male mice, the age-associated diminution in *Malat1* levels occured in adipocytes but not in the SVF (Fig 1C). Age did not modify the levels of *mascRNA* in the same cellular fraction (Fig 1D).

**Fig 2:**
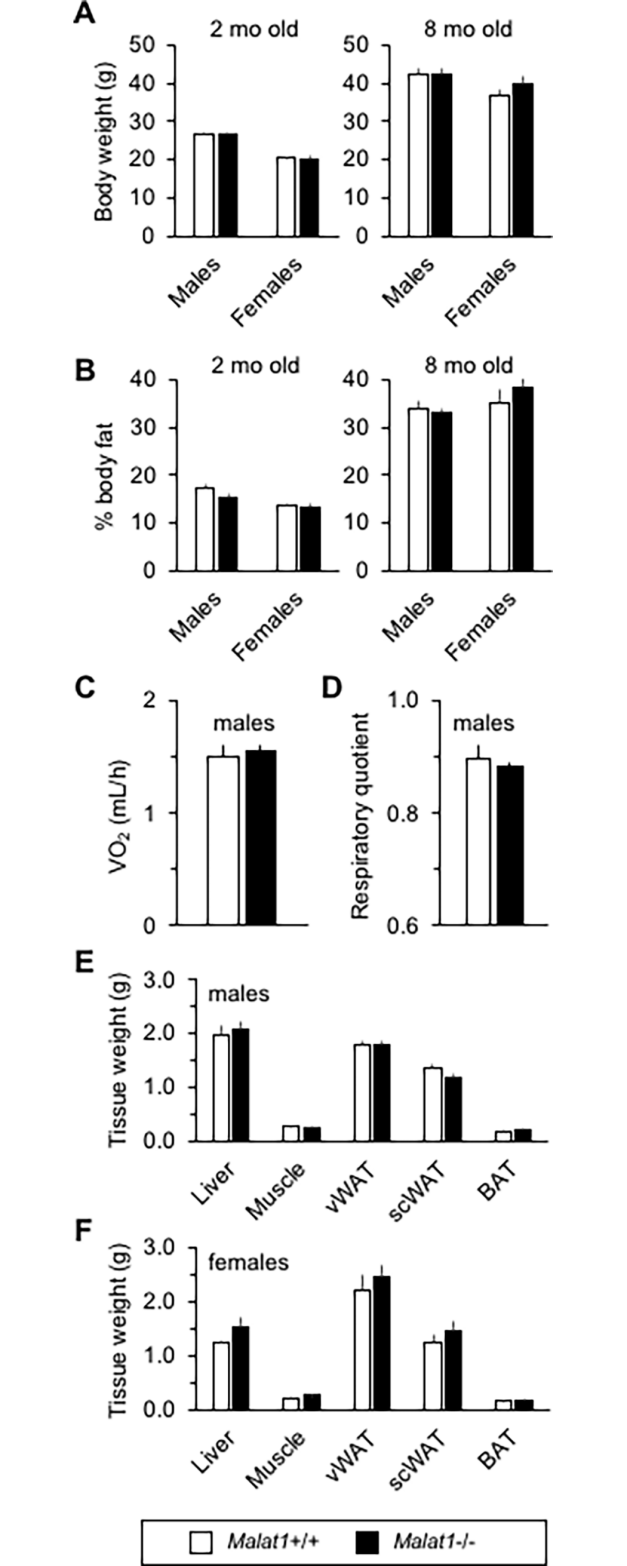
Absence of *Malat1* does not impact energy balance and body fat gain upon normal aging. (A) Body weight of male and female *Malat1*+/+ and *Malat1*-/- littermates at two (n = 10–15) and 8 months old (n = 6–12). All mice were fed regular chow. (B) Body fat percentage in male and female *Malat1*+/+ and *Malat1*-/- mice at two (n = 10–15) and eight months old (n = 6–11). (C) Oxygen consumption in male *Malat1*+/+ and *Malat1*-/- littermates at seven months old. (n = 5–6). (D) Respiratory quotient assessed by indirect calorimetry in mice described in C. (E-F) Weight of tissues harvested in male and female, eight months old *Malat1*+/+ and *Malat1*-/- mice. (males: n = 12 +/+, 10 -/-; females: n = 10 +/+, 6 -/-).

To assess the physiological relevance of a reduction in *Malat1* upon aging, we first documented age-associated changes in energy balance in *Malat1*+/+ and *Malat1*-/- littermates. Consistent with expected gene knockout in other tissues [16], *Malat1* transcripts were barely detectable in either vWAT or scWAT of *Malat1*-/- mice (S3 Fig). As reported, at a young age, male and female *Malat1*-/- mice had a body weight similar to their *Malat1*+/+ counterparts (Fig 2A). At eight months old, body weight was still similar between genotypes (Fig 2A). Consistently, no difference in body lean or fat mass (Fig 2B) or bone mineral density (data not shown) was observed between genotypes at either 2 or 8 months of life. The similar body weights in *Malat1*+/+ and *Malat1*-/- littermates were associated with an absence of difference in energy expenditure and respiratory quotient, at least in male mice (Fig 2C and 2D). At sacrifice, weights of white and brown adipose depots, liver, and skeletal muscle was also similar between *Malat1*+/+ and *Malat1*-/- male (Fig 2E) and female (Fig 2F) animals.

**Fig 3:**
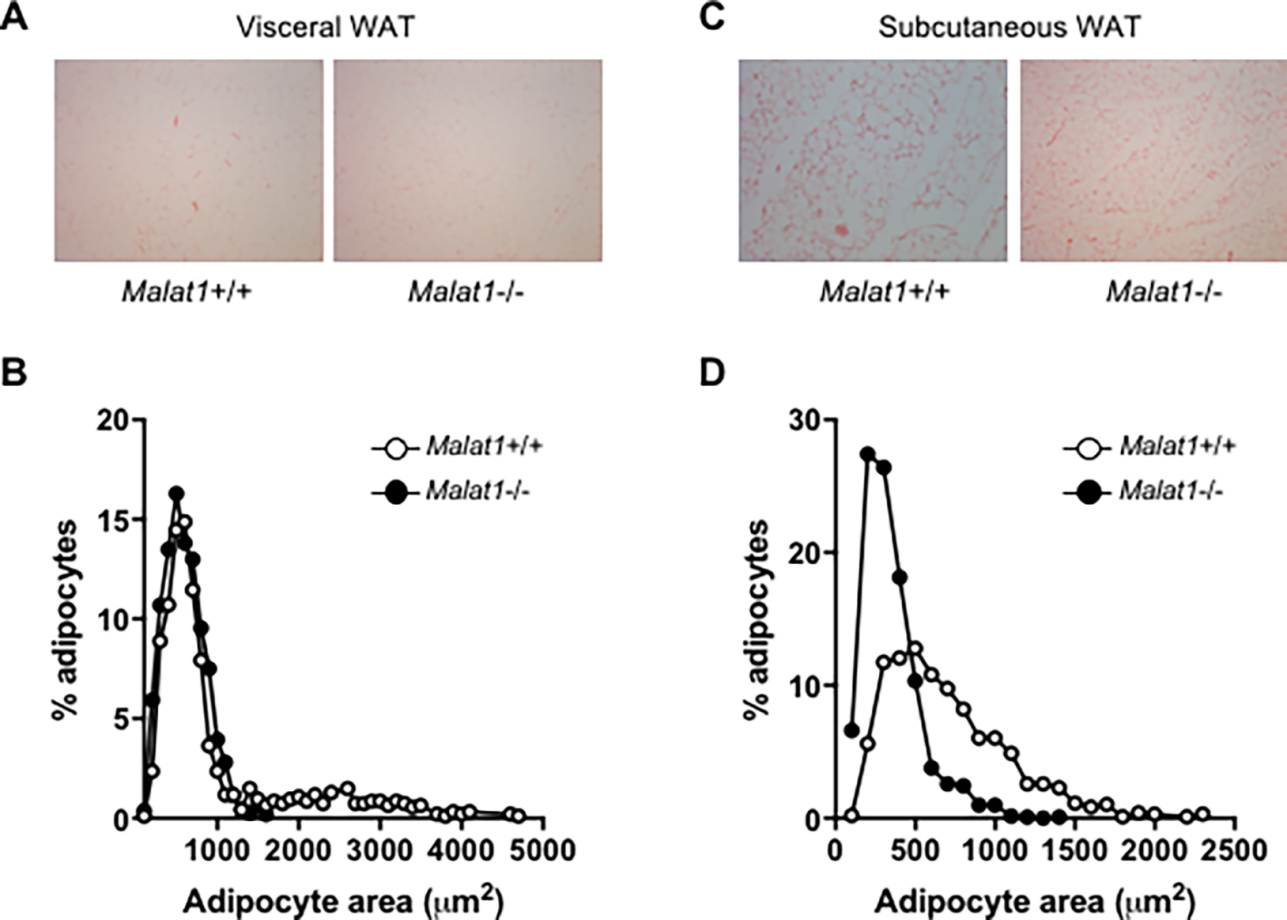
White adipose tissue histology in *Malat1*+/+ and *Malat1*-/- mice. Representative images of vWAT (A) and scWAT (C) and respective relative proportion of adipocytes as function of size (B-D). For adipocyte size, 934 and 1571 adipocytes in vWAT and 962 and 1328 in scWAT were analyzed within histological slides of two *Malat1*+/+ and 2 *Malat1*-/- 3 months old mice, respectively. In panels A and C, magnification is 200 X.

Despite the fact that the absence of *Malat1* did not modify the weight of WAT depots, the effects of this gene on adipocyte size and number in WAT depots were further documented since *Malat1* has been associated with proliferation/differentiation state transition. In vWAT, although both genotypes showed similar proportions of small adipocytes, we observed a higher prevalence of bigger adipocytes (more than 2000 μm^2^) in *Malat1*+/+ mice when compared to their *Malat1*-/- littermates (*p* < 0.0001, Fig 3A and 3B). Moreover, a striking shift in adipocyte size was also found in scWAT, where *Malat1*-/- mice exhibited higher numbers of much smaller adipocytes than *Malat1*+/+ mice (*p* < 0.0001, Fig 3C and 3D).

**Fig 4:**
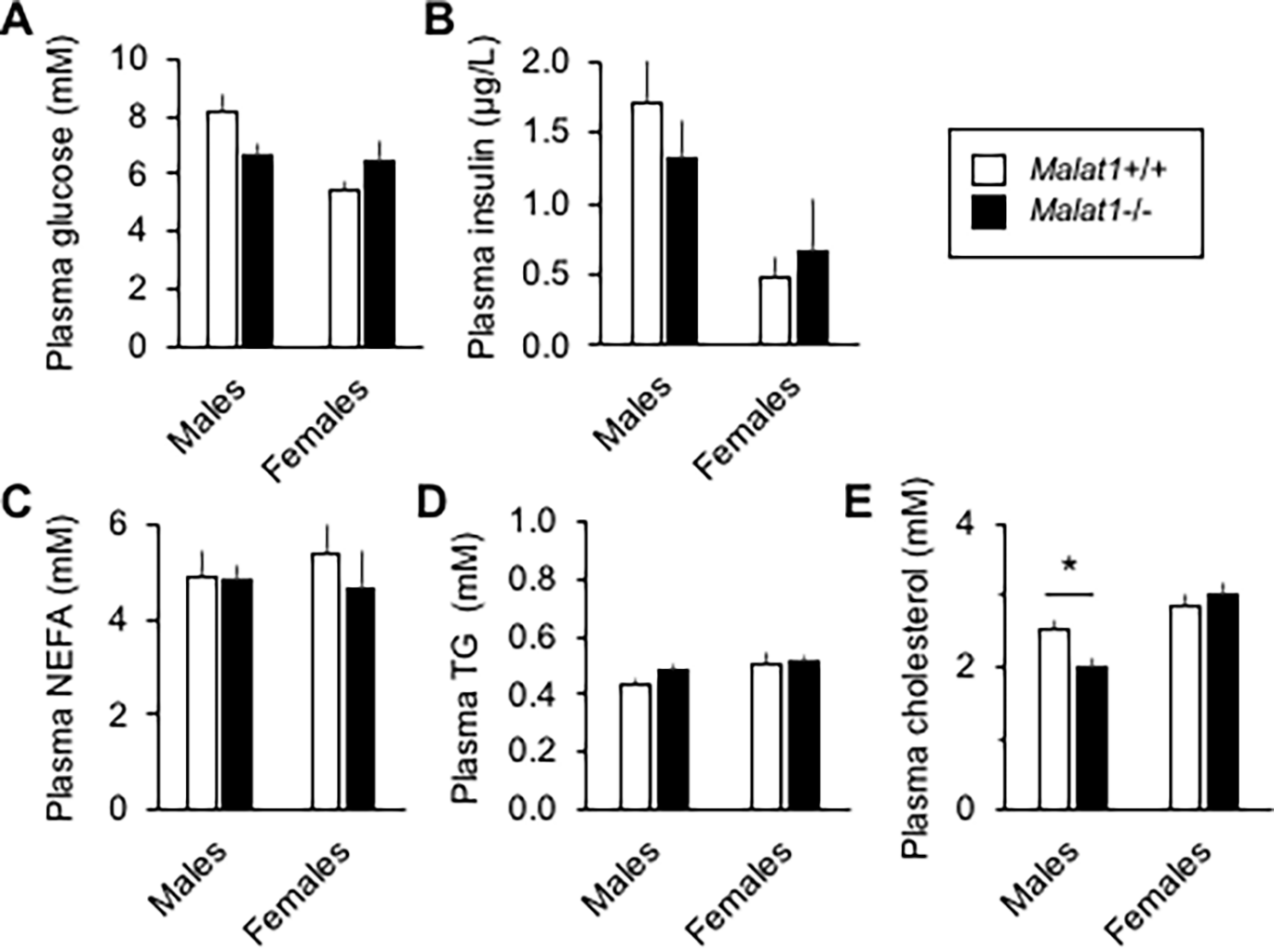
Glucose and lipid levels in 8 months old *Malat1*+/+ and *Malat1*-/- mice. Plasma levels of glucose (A), insulin (B), non-esterified fatty acids (NEFA) (C), triglycerides (D) and cholesterol (E) were quantified after overnight fasting. (n = 5–12). * indicates a significant difference compared with *Malat1*+/+ mice of the same gender (*p* < 0.05).

**Fig 5:**
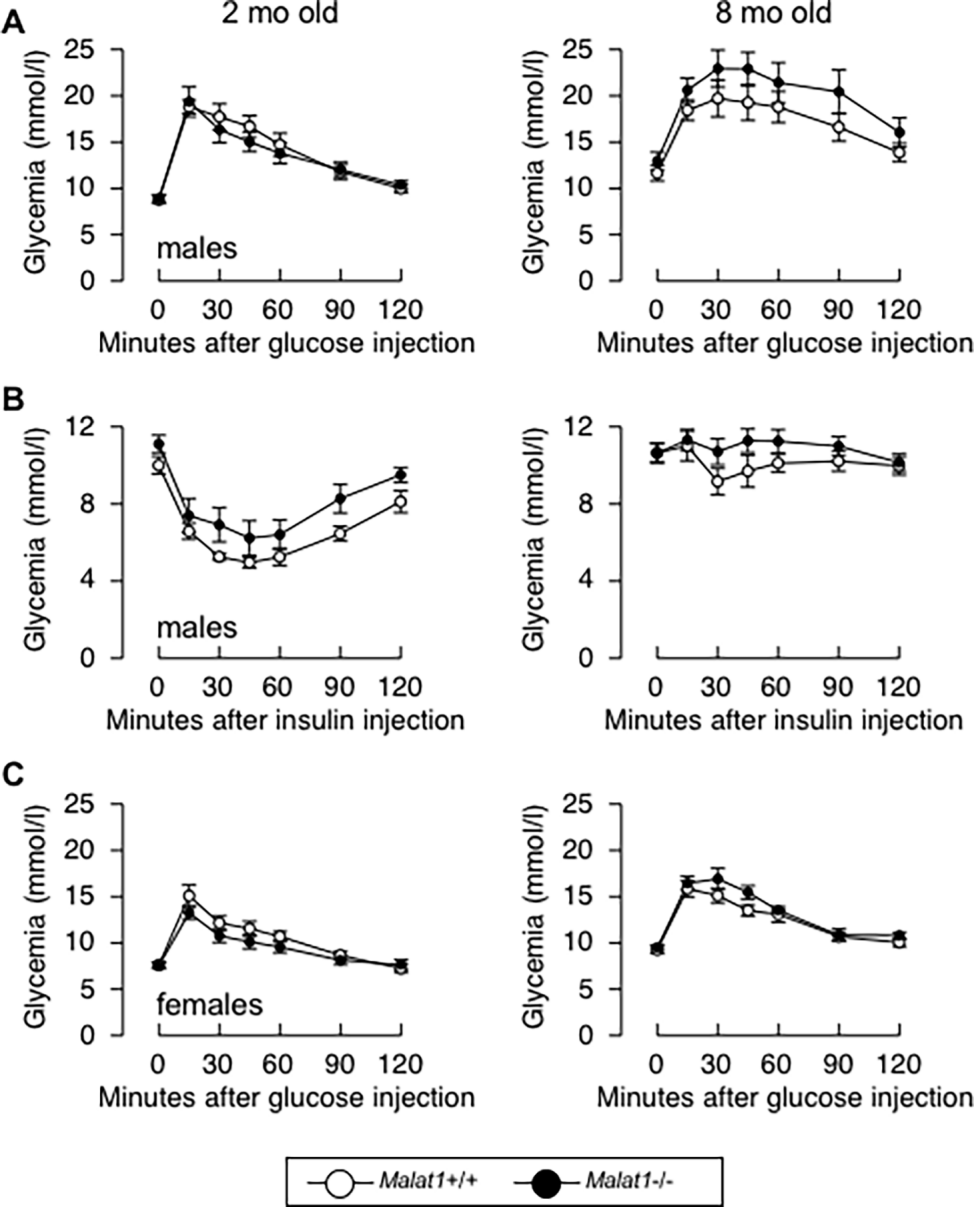
Absence of *Malat1* does not influence age-induced insulin resistance. (A) Glucose tolerance test in 2 (left) or 7.5 (right) months old male *Malat1*+/+ and *Malat1*-/- mice fed a chow diet. (n = 9–13). (B) Insulin sensitivity test in 2 (left) or 8 (right) months old male *Malat1*+/+ and *Malat1*-/- mice fed a chow diet. (n = 8–13). (C) Glucose tolerance test in 2 (left) or 7.5 (right) months old female *Malat1*+/+ and *Malat1*-/- mice fed a chow diet. (n = 6–10).

Because adipose tissue biology tightly modulates glucose and lipid metabolism, we next evaluated whether absence of *Malat1* impacts glycemia and lipid profiles in eight months old mice. We observed no difference between genotypes in the fasting levels of glucose and insulin (Fig 4A and 4B). Plasma levels of non-esterified fatty acids (NEFA) (Fig 4C) and triglycerides (Fig 4D) were also similar between *Malat1*+/+ and *Malat1*-/- mice. Whereas female *Malat1*+/+ and *Malat1*-/- mice had similar cholesterolemia, a small albeit significant lower level of cholesterol was found in *Malat1*-/- mice compared to their *Malat1*+/+ counterparts (Fig 4E). To further evaluate a possible link between *Malat1* and insulin resistance, glucose tolerance and insulin sensitivity tests were performed in *Malat1*+/+ and *Malat1*-/- male and female mice at a young and older age (2 vs 8 mo old). As expected, aging induced robust glucose intolerance and insulin resistance in both male and female animals (Fig 5A–5C, compare *Malat1*+/+ mice between ages). However, the absence of *Malat1* did not modify glucose tolerance (Figs 5A & 4C) or insulin sensitivity (Fig 5B) in response to aging, at least until the age of 8 months. These findings demonstrate that absence of *Malat1* does not attenuate nor accelerate age-induced fat gain and insulin resistance.

**Fig 6:**
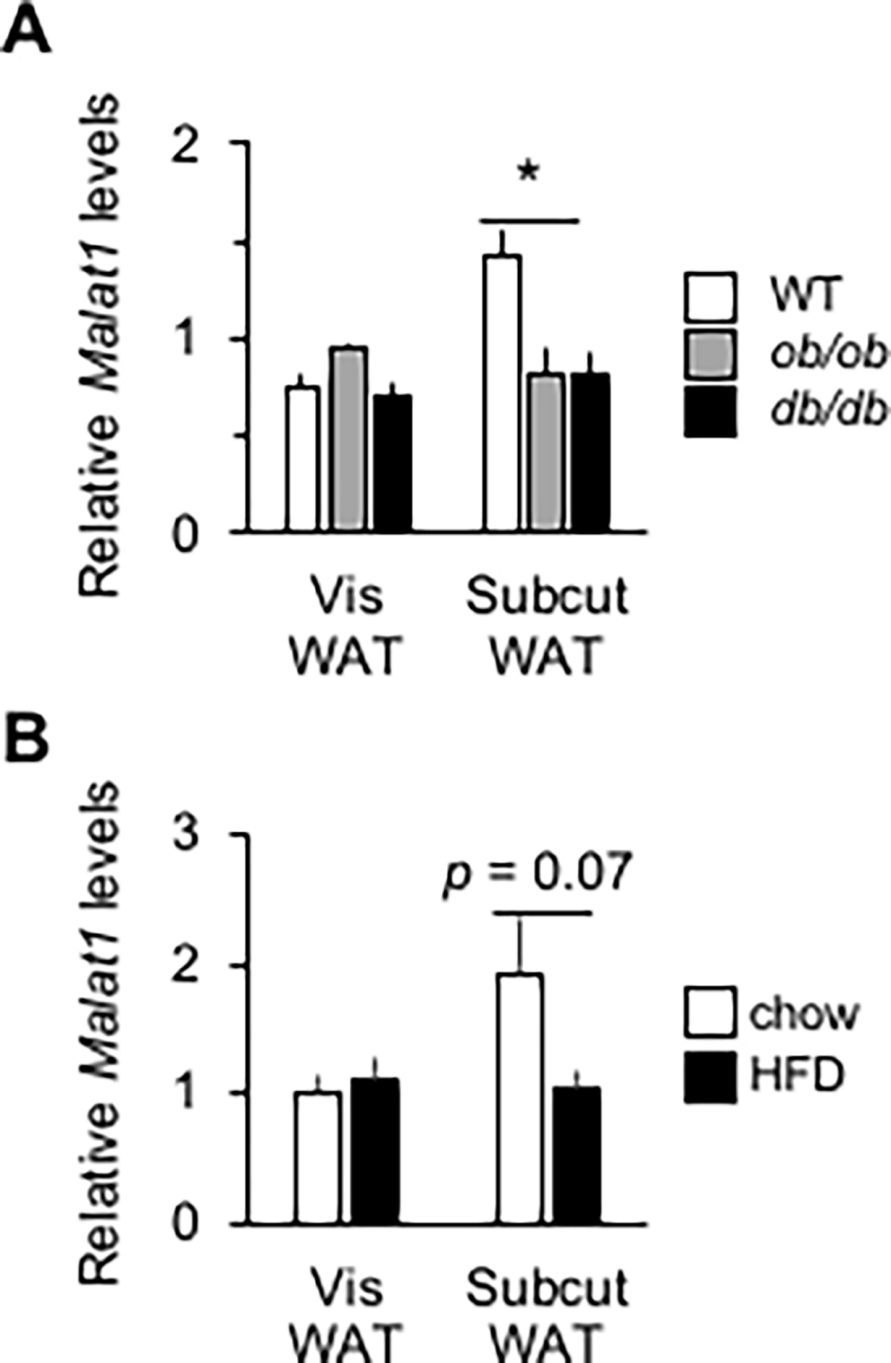
Obesity is associated with a reduction of *Malat1* levels in mice white adipose tissue. (A) *Malat1* expression levels in epididymal and inguinal WAT from two months old male *ob*/*ob*, *db*/*db* or their +/+ (WT) counterparts. (n = 8), * indicates a significant difference compared with the WT group (*p* < 0.05). (B) *Malat1* expression levels in epididymal and inguinal WAT from 6 months old male C57BL/6J mice fed a high-fat, high-sucrose diet for 4 months. (n = 9).

We next tested the hypothesis that, akin to our results in old mice (Fig 1), *Malat1* expression levels in WAT are also reduced in conditions of obesity. We observed that *Malat1* RNA levels in scWAT, but not vWAT, were significantly lower in leptin-deficient *ob/ob* obese mice as well as in leptin receptor-deficient *db/db* mice compared to those of their lean wild-type counterparts (Fig 6A). A similar trend (*p* = 0.07) was found in high-fat fed mice compared to their chow-fed controls (Fig 6B). Based on these results, we examined whether absence of *Malat1* would confer higher susceptibility to diet-induced fat accretion and insulin resistance.

**Fig 7:**
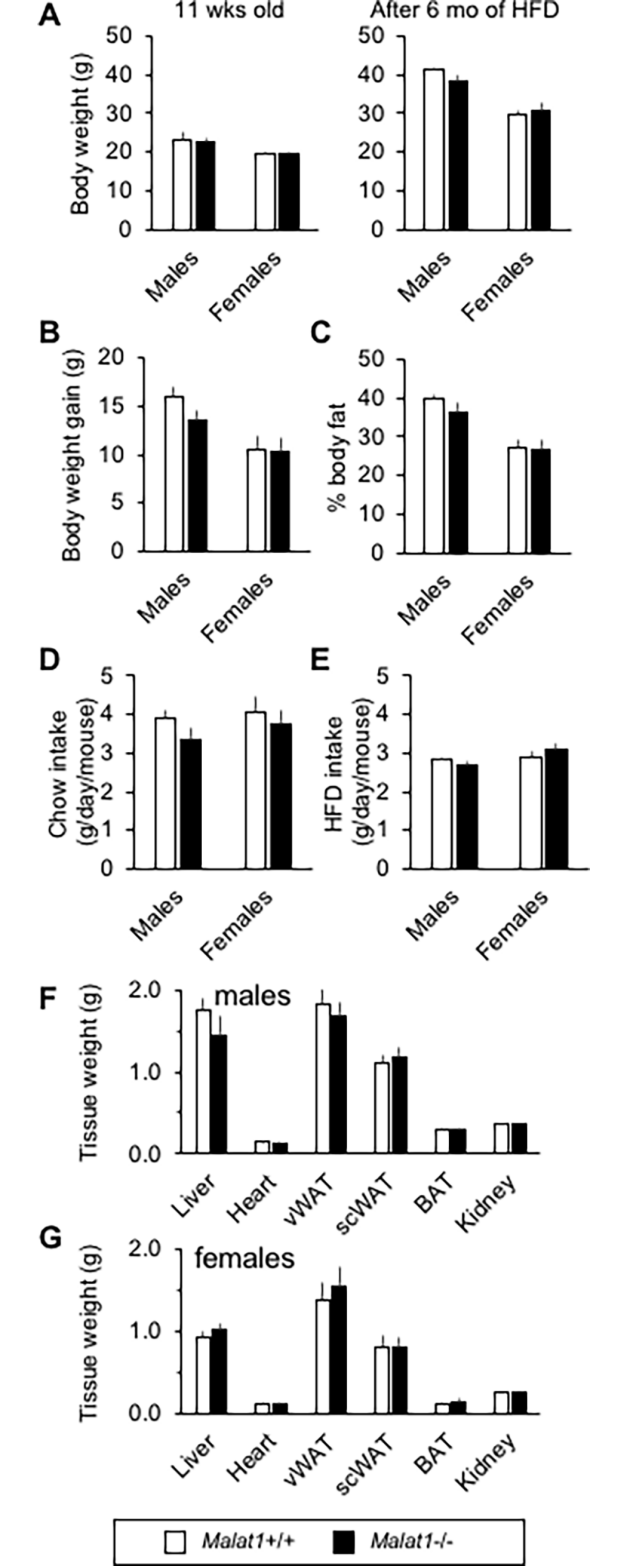
Absence of *Malat1* does not influence diet-induced obesity. (A) Body weight of male and female *Malat1*+/+ and *Malat1*-/- littermates fed regular chow until reaching 11 weeks old (left), and then placed on high-fat, high-sucrose diet for 6 months (right) (n = 8–10). (B) Body weight gain of male and female *Malat1*+/+ and *Malat1*-/- littermates during the first 10 weeks of high-fat, high-sucrose feeding regimen (n = 8–10). (C) Body fat percentage in male and female *Malat1*+/+ and *Malat1*-/- mice fed a high-fat, high-sucrose diet for two months (n = 6–8). (D) Daily food intake of male and female *Malat1*+/+ and *Malat1*-/- littermates fed regular chow. Data obtained the week before switching diet to a high-fat, high-sucrose diet (i.e. at 10 weeks old). (n = 8–10). (E) Daily food intake of male and female *Malat1*+/+ and *Malat1*-/- littermates fed a high-fat, high-sucrose diet. Data obtained 10 weeks after the beginning of the regimen (i.e. at 21 weeks old). (n = 8–10). (F-G) Weight of tissues harvested in male and female 9 months old *Malat1*+/+ and *Malat1*-/- mice fed a high-fat, high-sucrose diet since the age of 11 weeks. (males: n = 10 +/+, 9 -/-; females: n = 8 +/+, 8 -/-).

As found in the previous cohort (Fig 1A), *Malat1*+/+ and *Malat1*-/- mice had similar body weight before the beginning (eleven weeks old) of a high-fat (45%), high-sucrose (35%) diet (Fig 7A). Furthermore, although the diet itself induced very significant weight gain, we detected no difference in body weight between *Malat1*+/+ and *Malat1*-/- mice after up to 6 months of the feeding regimen (Fig 7A). Consistent with these results, body weight gain (Fig 7B) and body fat mass (Fig 6C) was also similar between genotypes, in both male and female mice. We found no difference between *Malat1*+/+ and *Malat1*-/- mice in daily food intake before (Fig 7D) or after (Fig 7E) the start of the diet. After 6 months on the high-fat, high-sucrose diet, the weights of white and brown adipose depots, liver, kidney, and the heart were similar between genotypes in both male (Fig 7F) and female (Fig 7G) mice. Thus, energy balance was not impacted by the absence of *Malat1* in the settings of diet-induced obesity.

**Fig 8:**
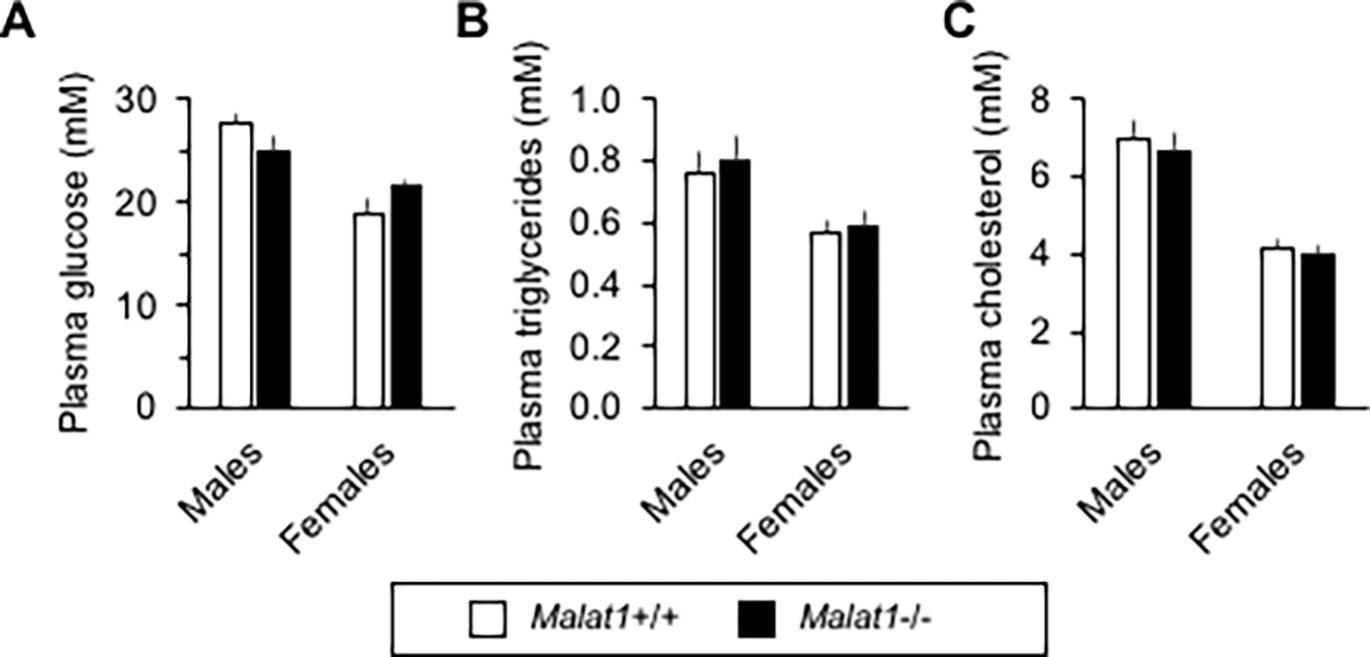
Glucose and lipid levels in diet-induced obese *Malat1*+/+ and *Malat1*-/- mice. Plasma levels of glucose (A), triglycerides (B) and cholesterol (C) were quantified after overnight fasting in male and female 9 months old *Malat1*+/+ and *Malat1*-/- mice fed a high-fat, high-sucrose diet since the age of 11 weeks. (n = 9–10).

**Fig 9:**
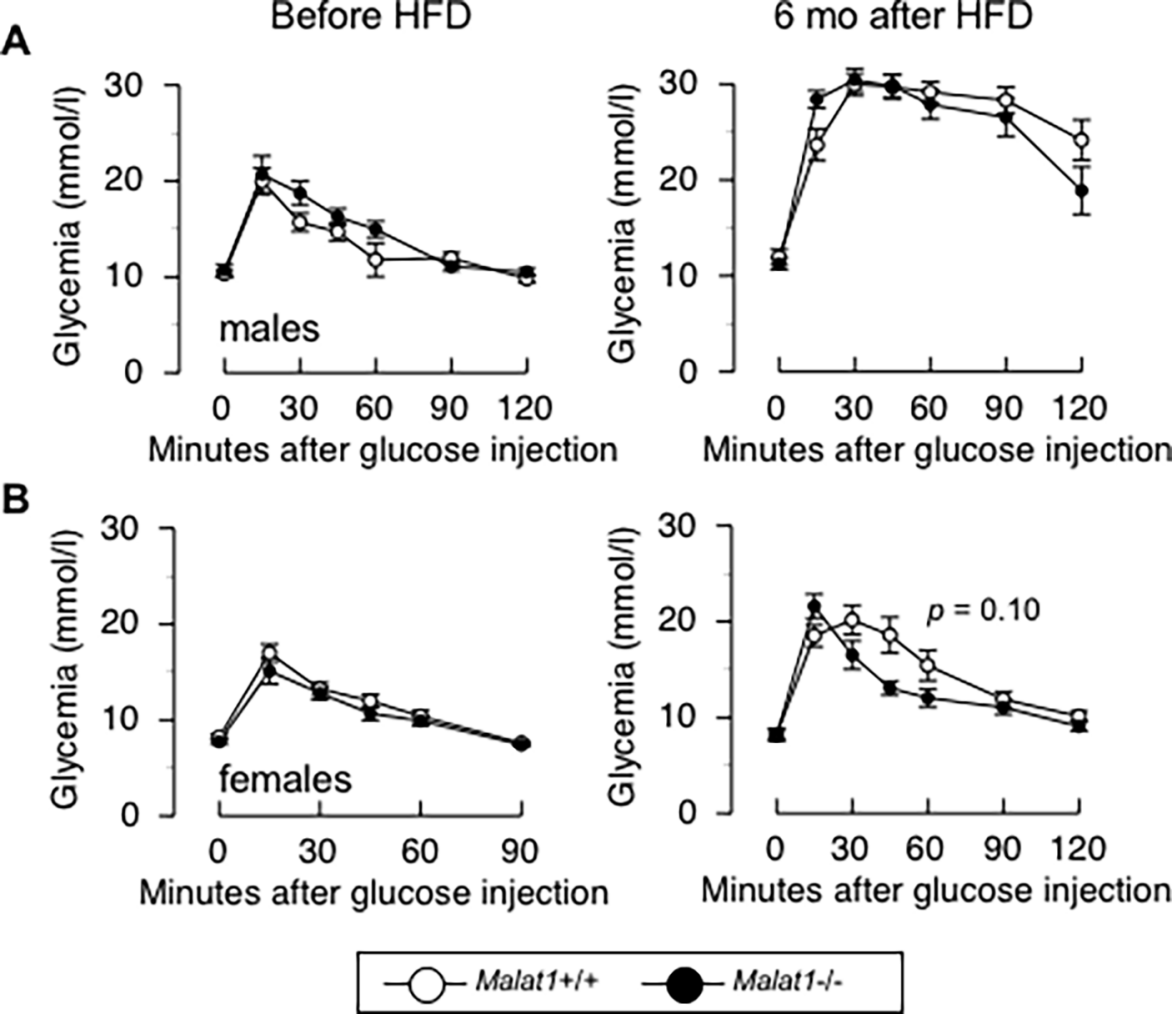
Absence of Malat1 does not influence diet-induced glucose intolerance. (A) Glucose tolerance test before (left) or after six months (right) of a high-fat, high-sucrose feeding regimen in male Malat1+/+ and Malat1-/- mice. (n = 6–10). (B) Glucose tolerance test before (left) or after six months (right) of a high-fat, high-sucrose feeding regimen in female Malat1+/+ and Malat1-/- mice. (n = 7–8).

No difference between *Malat1*+/+ and *Malat1*-/- high-fat, high-sucrose fed mice were observed in the fasting levels of glucose (Fig 8A), triglycerides (Fig 8B), or cholesterol (Fig 8C), in either male or female animals. Confirming our previous findings, there was no significant impact of *Malat1* absence on glucose tolerance in male (Fig 9A) and female (Fig 9B) before the beginning of the feeding regimen. Whereas the diet itself induced very significant glucose intolerance (compare glycemic responses between *Malat1*+/+ mice before and after 6 months on the diet), we detected no difference in glucose excursions between *Malat1*+/+ and *Malat1*-/- mice after up to 6 months of the feeding regimen. Taken together, these findings demonstrate that absence of *Malat1* does not attenuate nor accelerate diet-induced fat gain and insulin resistance.

## Discussion

Early studies using cDNA microarrays in aging mice have shown important changes in genomic profiles in several tissues. These are partially reversed by calorie restriction (CR), an established regimen for prolonging longevity, while reducing incidence and severity of a myriad of age-associated diseases [26–30]. In epididymal vWAT of aged mice, long-term CR increases the expression of genes involved in insulin sensitization and carbohydrate metabolism [31] and reduces that of genes involved in inflammation [32]. However, because of their inherent nature, these cDNA microarrays were limited in their ability to detect and evaluate expression of non-coding RNA. In the present study, we used the RAP-PCR method on vWAT and discovered that, among other genes, the expression level of the lncRNA *Malat1* was consistently diminished in aged mice and men. However, contrary to our hypothesis, engineered genetic deletion of *Malat1* in mice did not influence fat gain or glucose tolerance upon aging (at least until 8 months old) nor in the context of diet-induced obesity.

*Malat1* is conserved between mouse and human [33, 34] and its tissue expression is ubiquitous, albeit highest expression levels are found in the lungs [16, 19, 33]. *Malat1* is highly expressed in nuclear speckles [33], especially upon cellular state of active gene transcription such as proliferation [35]. Indeed, *in vitro* experiments have demonstrated the stimulating impact of *Malat1* on cell division [10, 14, 36]. Interestingly, since the function of progenitor cells and replication of pre-adipocytes decline during aging [2], it is possible that a reduction of *Malat1* expression contributes to this phenomenon, although the present study did not specifically address this issue. The age-associated reduction in *Malat1* expression in WAT is thus intriguing. *Malat1* is a putative target gene of FoxP3 [37], a downstream effector of mechanistic target of rapamycin mTOR [38]. However, mTOR is more, not less active in adipose tissue of aged mice [39], which in theory should increase *Malat1* expression. *Malat1* transcription is also under the control of the Hypoxia-inducible factor 1-alpha (HIF-1-alpha) [40, 41]; however, an increase, not a decrease, in hypoxia is observed during aging, especially in vWAT [42]. This indicates that the reduction in *Malat1* levels in aged mice and men perhaps occurs in spite of these two possible stimulating pathways.

It is noteworthy that in old female mice as well as in models of obesity, *Malat1* expression was downregulated in scWAT rather than in vWAT. These findings suggest that *Malat1* RNA levels are under the modulation of sex hormones, which are greatly modulated in obesity [43]. It is also likely that, although both conditions are characterized by fat accumulation and insulin resistance, obesity drives different molecular cues than aging [44–46]. Moreover, reduced levels of *Malat1* could be due to lower transcription rates and/or increased RNA instability upon aging [47, 48], although the lack of age-induced changes in *mascRNA* indicates that the latter might not represent the main mechanism. Interestingly, a recent study performed in human tissues found a new *MALAT1* spliced variant [49]. Whether mice also express this transcript in adipose tissue, and whether its levels also decline upon aging or rather increased in compensation was not studied herein. Thus, the exact molecular pathways that contribute to the age-associated reduction in *Malat1* in WAT remain to be experimentally unraveled.

Previous studies have demonstrated that *Malat1* ablation in mice has little impact on development, and that adult *Malat1*-/- animals housed under normal husbandry conditions show no obvious phenotype [16, 17, 19, 20]. This suggests that *Malat1* is nonessential and/or redundant with other lncRNAs. A clear limitation of these embryonic models is that compensatory mechanisms are likely activated early in life; as such, effects of *Malat1* deletion could perhaps be detected in an inducible model. Akin to several other mouse models, it was furthermore postulated that proliferative or metabolic challenges might better reveal a physiological role for *Malat1* [18]. Yet, our results clearly demonstrate that deletion of *Malat1* has no significant effect–either stimulation or inhibition–on age- or diet-induced changes in body weight, body fat accretion, and glucose and lipid metabolism. Male and female *Malat1*-/- mice gained as much weight as their *Malat1*+/+ littermates when studied up to 8 and 9 months old on regular chow or a high-fat, high-sucrose diet, respectively. Coherent with a steady energy balance, oxygen consumption and food intake were similar between *Malat1*+/+ and *Malat1*-/- mice. Whether this indicates a minimal role of *Malat1* in the brain and/or compensatory response to altered levels of satiety signals from the periphery remains to be explored. These findings thus nullify our initial hypothesis suggesting that if Malat1 expression declines with age, and if Malat1 were a regulatory node, a genetically-engineered deletion of Malat1 by itself should result in premature development of age-associated metabolic disorders. However, it is possible that the reduction in *Malat1* during aging occurs at the same time than other age-associated changes, and that the interactions between these processes are required to affect energy metabolism. This concept should be tested in future projects.

Our group previously found that absence of *Malat1* favored higher cholesterolemia in a model of drug-induced hepatocarcinogenesis [16]. In contrast, in the present study, male (but not female), eight months-old *Malat1*-/- mice had slightly but significantly lower plasma cholesterol levels than *Malat1*+/+ animals. This effect was observed in the aging cohort fed regular chow, but not in the cohort fed a high-fat diet, which induced hypercholesterolemia similarly in both *Malat1*+/+ and -/- mice. Interestingly, it was shown that *Malat1* expression is enhanced in hepatocytes of obese *ob/ob* mice, and that it promotes cholesterol synthesis by stabilizing SREBP-1c protein activity [15]. Moreover, oligonucleotide-based knowndown of *Malat1* reduced hepatic cholesterol content in *ob/ob* animals [15]. Our recent findings associating *Malat1* deletion and lower circulating cholesterol levels are thus consistent with the latter study. More work is however required to better understand whether *Malat1* impacts circulating lipoprotein metabolism besides its effects on cholesterol production in the liver.

In our experimental settings, deletion of *Malat1* had no significant effect on glucose tolerance and insulin sensitivity in either contexts of aging or obesity. Consistent with a recent paper by Chen et al. [50], no differences were found in fasting glycemia and insulinemia between *Malat1*+/+ and *Malat1*-/- levels. However, this paper also reported enhanced insulin sensitivity and insulin secretion in Malat1-/- mice upon glucose injection and refeeding [50], which we did not observe. Discrepancies between the two studies are possibly due to differences in the length of fasting before glucose and insulin injections, or husbandry conditions, notably animal facility microbiota. It is also possible that more refined methods such as the euglycemic-hyperinsulinemic clamp could have better discriminate subtle effects of *Malat1*. Yet, our observations suggest that production of insulin or its immediate signaling via the insulin receptor are likely not defective or enhanced in the absence of *Malat1*.

Adipocyte hyperplasia and hypertrophy play important roles in the modulation of insulin sensitivity, and impaired regulation of these processes has been reported in aging and obesity [2, 51]. *Malat1* has been linked to cell proliferation in several *in vitro* models, and *Malat1*-/- mice showed significantly increased adipocyte numbers per depot, suggesting enhanced adipogenesis. However, these cells were smaller in size, which resulted in similar fat pad weights between *Malat1*-/- mice and their *Malat1*+/+ littermates. We note that these differences in adipocyte size and number were not reflected by a reciprocal impact on whole-body insulin sensitivity. This is surprising, since higher numbers of small adipocytes are typically linked with insulin sensitization effects [1]. It is thus possible that besides a theoretically more insulin-sensitive WAT, other tissues have developed insulin resistance in the absence of *Malat1*, although this would require additional experimental characterization.

Therefore, in light of the present findings, we conclude that the overall metabolic impact of the absence of *Malat1* on adipose tissue accretion and glucose intolerance is either physiologically not relevant upon aging and obesity, or that it is masked by as yet unknown compensatory mechanisms. Because we used a germinal, whole-body knockout of the *Malat1* gene, a thorough analysis of tissue-specific effects of *Malat1* using targeted and inducible models could perhaps help uncover its roles. It is also plausible that challenges/conditions other than aging and diet-induced obesity could best highlight the physiological and molecular actions of *Malat1*.

## Supporting information

S1 Table Primers used for RAP-PCR. (DOCX)

S2 Table Primers used for qPCR. (DOCX)

S3 Table Genes with marked visual differences in expression levels as evaluated by autoradiography after RAP-PCR performed on visceral white adipose tissue of aging male C57BL/6J mice using the strategy described in S1 Fig. The *p* value indicates the validation by qPCR of a significant difference in mRNA levels in vWAT of 4, 12 or 24 months old male C57BL/6J mice (n = 12–14), tested by one-way ANOVA. NS: no significant change after quantification by qPCR; ND: not determined.
(DOCX)

S1 Fig Description of the gene discovery strategy A. RAP-PCR was used on epididymal adipose tissue of male C57BL/6J mice aged 4, 12 and 24 months. Bands differentially expressed between age groups were visualized on an autoradiogram, excised from the gel, re-amplified, isolated, cloned, and sequenced. B. After removal of duplicates, genes were selected on the basis of novelty and extent of modulation upon aging. Variation in expression for the chosen genes was then confirmed by qPCR in adipose tissue from mice of a second cohort, and in adipose tissue from men.
(TIFF)

S2 Fig (A) Expression levels of *Malat1* in gonadal (visceral—vWAT) and inguinal (subcutaneous—scWAT) white adipose tissue of 4, 12 or 24 months old female C57BL/6J mice (n = 5). * indicates a significant difference compared with the 4 months old group (p < 0.05). (B) *Malat1* expression in in cells of the stroma vascular fraction (SVF) or adipocyte freshly collagenase-isolated from visceral or subcutaneous of young male mice (n = 3). (C) *Malat1* expression in in cells of the stroma vascular fraction (SVF) or adipocyte freshly collagenase-isolated from visceral or subcutaneous of young men (n = 3). * indicates a significant difference compared with the respective SVF group (p < 0.05).
(TIFF)

S3 Fig Expression levels of *Malat1* in epididymal visceral WAT and inguinal subcutaneous WAT in Malat1+/+ and Malat1-/- male mice. n = 6. p < 0.0001 for both tissues. In *Malat1*-/- mice, levels were barely detectable by qPCR.
(TIFF)
