# Supplementary material for: Loss of Malat1 does not modify age- or diet-induced adipose tissue accretion and insulin resistance in mice
Source: PLoS One. 2018 May 10;13(5):e0196603. doi: 10.1371/journal.pone.0196603 (PMC5944987; doi:10.1371/journal.pone.0196603)
Supplement: S1 Table — (DOCX) [file pone.0196603.s001.docx]

**Supplementary Table 1.**

| A1 | AATCTAGAGCTCCTCCTC |
| --- | --- |
| A2 | AATCTAGAGCTCCAGCAG |
| A3 | AATCTAGAGCTCTCCTGG |
| A4 | AATCTAGAGCTCTCCAGC |
| A5 | AATCTAGAGCTCCCTCCA |
