# Supplementary material for: Loss of Malat1 does not modify age- or diet-induced adipose tissue accretion and insulin resistance in mice
Source: PLoS One. 2018 May 10;13(5):e0196603. doi: 10.1371/journal.pone.0196603 (PMC5944987; doi:10.1371/journal.pone.0196603)
Supplement: S2 Table — (DOCX) [file pone.0196603.s002.docx]

**Supplementary Table 2.**

|  | Forward | Reverse |
| --- | --- | --- |
| Mouse *Malat1* | CTTTGCGGGTGTTGTAGGTTT | GAGGCTTGTGGTAGGTCATCTGTT |
| Mouse *mascRNA* | TGGTTTCCAGGACGGGGTTCA | TGGAGACACCGCAGGGACTTGA |
| Human *MALAT1* | GTAATGGAAAGTAAAGCCCTGAAC | CCCCGGAACTTTTAAAATACCTCT |
| Human *mascRNA* | TGGTTTCCAGGACGGGGTTCAA | TGGAGACGCCGCAGGGATTT |
| Mouse β-actin | CTCTAGACTTCGAGCAGGAG | AGAGTACTTGCGCTCAGGAG |
| Human L27 | GTCGGGCCTTGCGTTTAAG | CACCTAATGCCCACAAGGTACTC |
