# Supplementary material for: Loss of Malat1 does not modify age- or diet-induced adipose tissue accretion and insulin resistance in mice
Source: PLoS One. 2018 May 10;13(5):e0196603. doi: 10.1371/journal.pone.0196603 (PMC5944987; doi:10.1371/journal.pone.0196603)
Supplement: S3 Table — The p value indicates the validation by qPCR of a significant difference in mRNA levels in vWAT of 4, 12 or 24 months old male C57BL/6J mice (n = 12–14), tested by one-way ANOVA. NS: no significant change after quantification by qPCR; ND: not determined. (DOCX) [file pone.0196603.s003.docx]

**Supplementary Table 3**

| **Up-regulated upon aging** | | | **Down-regulated upon aging** | | |
| --- | --- | --- | --- | --- | --- |
| **Genebank** | **Name** | **Validation by qPCR**  ***p* value** | **Genebank** | **Name** | **Validated by qPCR** |
| NM_001081308.1 | Taok3 | NS | EF177380.1 | Malat1 | 0.0138 see Figure 1A |
| NM_172586.2 | Zfp322a | 0.0262 | NM_025799.4 | Fuca2 | NS |
| NM_133801.2 | Gtf2f1 | NS | AY542490.1 | KUNITZ5 | ND |
| NM_172900.2 | Siglecg | ND | NM_175678.3 | Npsr1 | ND |
| NW_001030529.1 | MEF2C | NS | D14571.1 | PEBP2b2 | NS |
| NM_001034964.1 | Sorbs1 | ND | AL591127.12 | Dnttip1 | ND |
| AB119275.1 | GATA6 | ND | NM_016813.2 | Nxf1 | NS |
| AL607084.14 | Park7 | NS | J04953.1 | Gelsolin | NS |
| AL928582.5 | Tbl1x | NS | NM_016918.3 | Nudt5 | ND |
| NM_133985.1 | Oxsr1 | NS | NM_008410.2 | Itm2b | NS |
| NM_011330.3 | Ccl11 | <0.0001 | NM_026242.3 | Mrfap1 | 0.05 |
| NM_001039530.3 | Parp14 | 0.0071 | NM_181324.2 | DEAD ((Asp-Glu-Ala-Asp) box polypeptide 6 (Ddx6)) | 0.0009 |
