# Supplementary figures and images for: Loss of Malat1 does not modify age- or diet-induced adipose tissue accretion and insulin resistance in mice
Source: PLoS One. 2018 May 10;13(5):e0196603. doi: 10.1371/journal.pone.0196603 (PMC5944987; doi:10.1371/journal.pone.0196603)

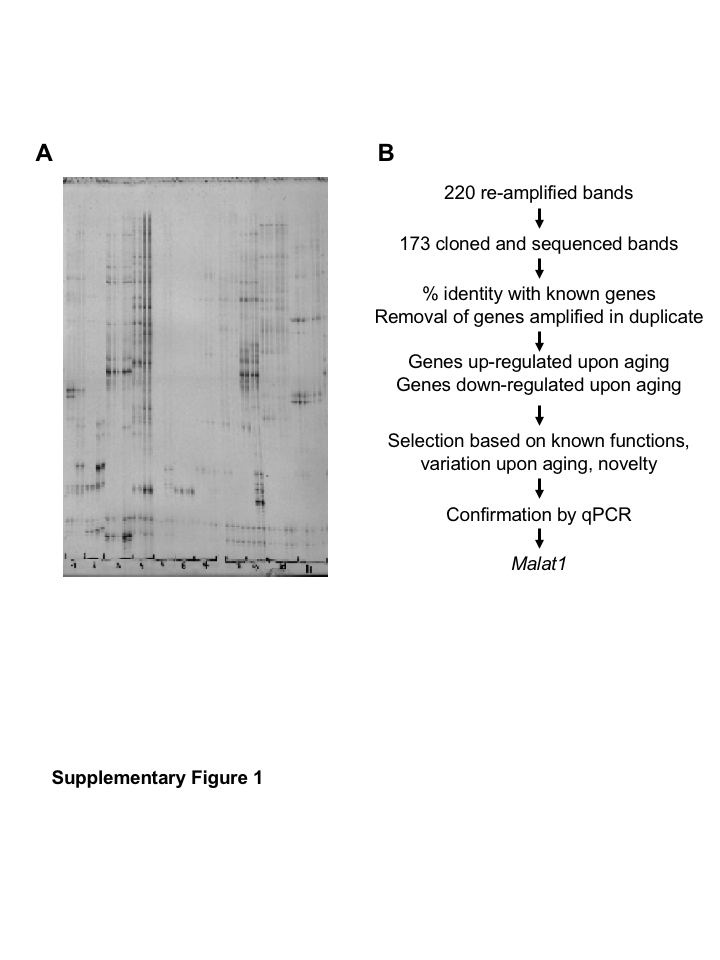

Supplement: S1 Fig — Description of the gene discovery strategy A. RAP-PCR was used on epididymal adipose tissue of male C57BL/6J mice aged 4, 12 and 24 months. Bands differentially expressed between age groups were visualized on an autoradiogram, excised from the gel, re-amplified, isolated, cloned, and sequenced. B. After removal of duplicates, genes were selected on the basis of novelty and extent of modulation upon aging. Variation in expression for the chosen genes was then confirmed by qPCR in adipose tissue from mice of a second cohort, and in adipose tissue from men. (TIFF) [file pone.0196603.s004.tiff]

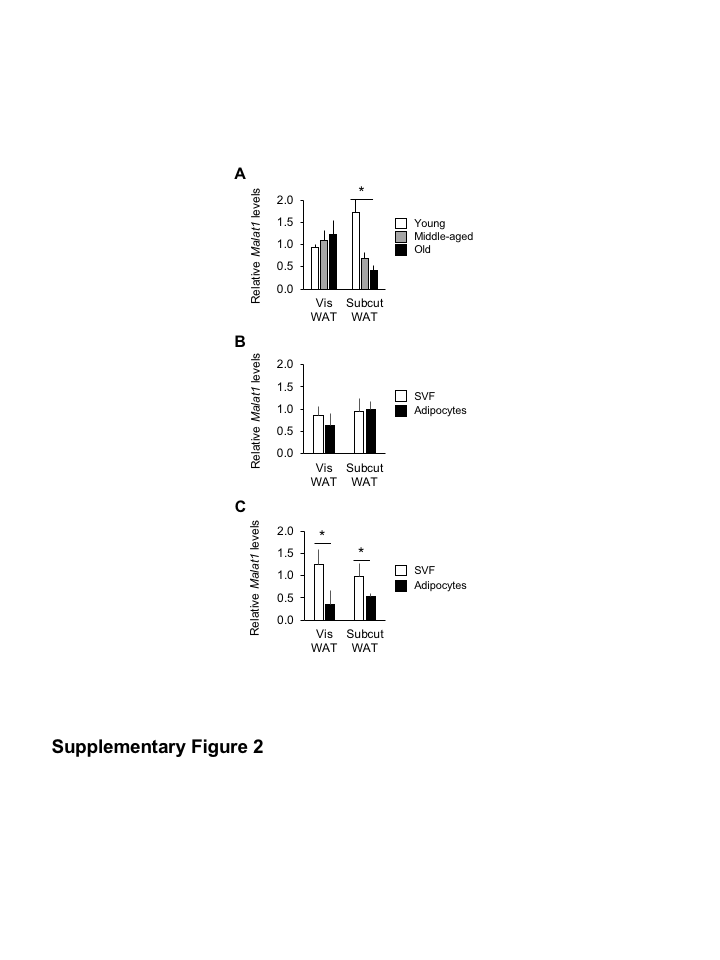

Supplement: S2 Fig — (A) Expression levels of Malat1 in gonadal (visceral—vWAT) and inguinal (subcutaneous—scWAT) white adipose tissue of 4, 12 or 24 months old female C57BL/6J mice (n = 5). * indicates a significant difference compared with the 4 months old group (p < 0.05). (B) Malat1 expression in in cells of the stroma vascular fraction (SVF) or adipocyte freshly collagenase-isolated from visceral or subcutaneous of young male mice (n = 3). (C) Malat1 expression in in cells of the stroma vascular fraction (SVF) or adipocyte freshly collagenase-isolated from visceral or subcutaneous of young men (n = 3). * indicates a significant difference compared with the respective SVF group (p < 0.05). (TIFF) [file pone.0196603.s005.tiff]

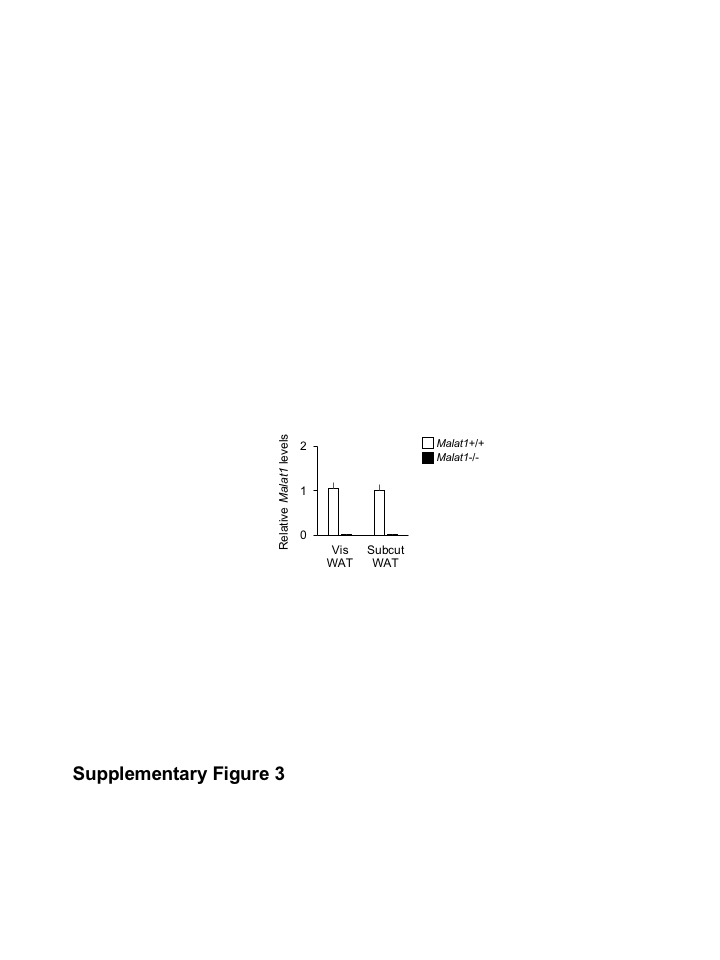

Supplement: S3 Fig — n = 6. p < 0.0001 for both tissues. In Malat1-/- mice, levels were barely detectable by qPCR. (TIFF) [file pone.0196603.s006.tiff]
